# PRO-INFLAMMATORY CYTOKINES IN OLDER AFRICAN AMERICAN WOMEN IN A PAIN AND DEPRESSION INTERVENTION

**DOI:** 10.1093/geroni/igae098.3694

**Published:** 2024-12-31

**Authors:** Rebekah Bhansali, Melissa Hladek, Catherine Clair, Sarah Szanton, Jessica Gill, Janiece Taylor

**Affiliations:** Johns Hopkins University, Severn, Maryland, United States; Johns Hopkins University, Johns Hopkins University, Maryland, United States; Johns Hopkins Bloomberg School of Public Health, Baltimore, Maryland, United States; Johns Hopkins University, Baltimore, Maryland, United States; Johns Hopkins University, Baltimore, Maryland, United States; Johns Hopkins University, Johns Hopkins University, Maryland, United States

## Abstract

Aging, pain, and depression are interconnected health concerns that significantly impact older women. African American women face additional risks of comorbid pain and depression, severe depressive symptoms, and less frequent pain treatment. Pro-inflammatory cytokines and inflammatory mediators are associated with both conditions. The DAPPER (Depression and Pain Perseverance through Empowerment and Recovery) pilot study tailored nurse-led interventions to meet the needs of older African American women with chronic pain and depression. This pilot study assessed non-pharmacological interventions among African American women aged 50+ with chronic pain and depressive symptoms. The intervention consisted of eight nurse-led sessions. Participants were randomized into waitlist control and immediate intervention groups. Ten participants in the immediate intervention group completed the program and provided pre- and post-intervention saliva samples. Outcomes, including goal attainment, pain, depression, and stress, were measured using validated tools such as PROMIS-8 and PSS-14. Saliva samples were collected for cytokine analysis, including TNFα, IL-8, IL-6, and IL-1β. Although under-powered for direct conclusions, data shows a downward trend in psychosocial and pro-inflammatory biomarkers. Pre- to post-intervention mean levels of psychosocial and cytokine markers (TNFα, IL-8, IL-6, IL-1β) decreased in a subset of the immediate intervention group, supported by median sensitivity analyses. The DAPPER pilot study acknowledged the intersectionality of race, age, and gender to provide personalized care and considered the biochemical mechanisms involved in pain and depression. Further research is needed to explore the potential biochemical impact of nurse-led, home-based interventions, particularly for older African American women.

